# Influence of Vitamin D on the Incidence of Metabolic Syndrome and Hormonal Balance in Patients with Polycystic Ovary Syndrome

**DOI:** 10.3390/nu15132952

**Published:** 2023-06-29

**Authors:** Katarzyna Lejman-Larysz, Anna Golara, Marta Baranowska, Mateusz Kozłowski, Paweł Guzik, Iwona Szydłowska, Jolanta Nawrocka-Rutkowska, Elżbieta Sowińska-Przepiera, Aneta Cymbaluk-Płoska, Agnieszka Brodowska

**Affiliations:** 1 Department of Gynecology, Endocrinology and Gynecological Oncology, Pomeranian Medical University in Szczecin, Unii Lubelskiej 1, 71-252 Szczecin, Poland; kmlejman@gmail.com (K.L.-L.); iwonaszyd@wp.pl (I.S.); jolanaw@poczta.onet.pl (J.N.-R.); agabrod@wp.pl (A.B.); 2 Department of Reconstructive Surgery and Gynecological Oncology, Pomeranian Medical University in Szczecin, Al. Powstańców Wielkopolskich 72, 70-111 Szczecin, Poland; marciabaranowska2211@gmail.com (M.B.); mtkoozo@gmail.com (M.K.); aneta.cymbaluk@gmail.com (A.C.-P.); 3 Clinical Department of Gynecology and Obstetrics, City Hospital, 35-241 Rzeszów, Poland; pawelguzik@gmail.com; 4 Department of Endocrinology, Metabolic and Internal Diseases, Pomeranian Medical University in Szczecin, Unii Lubelskiej 1, 71-252 Szczecin, Poland; elzbieta.sowinska.przepiera@pum.edu.pl

**Keywords:** vitamin D, polycystic ovary syndrome, metabolic syndrome, sex-hormone-binding globulin, prolactin, thyroid-stimulating hormone, androstendione, dehydroepiandrosterone sulfate, follicle-stimulating hormone, luteinizing hormone

## Abstract

Polycystic ovary syndrome (PCOS) is a heterogeneous endocrine disorder that affects 8–13% of women of reproductive age. It is one of the most common causes of infertility and is associated with hyperandrogenism in the form of hirsutism and acne, non-ovulatory cycles, and characteristic ovarian morphology. The available research on serum vitamin D deficiency in patients with PCOS and the appropriateness of vitamin D supplementation in this group of women is inconclusive, so we decided to investigate the influence of vitamin D on the incidence of metabolic syndrome and hormonal balance in patients with polycystic ovary syndrome. The study comprised 120 women aged between 18 and 42 years, who were divided into two groups: a group with diagnosed polycystic ovary syndrome (PCOS) and a group of regularly menstruating women without features of androgenisation, in whom polycystic ovary syndrome was excluded. Each patient underwent a history and physical examination, including a gynecological examination, anthropometric measurements were taken, including height, weight, waist, and hip circumference, and blood pressure was measured using the Korotkow method. In the female patients, the following parameters were also determined from the blood: follicle-stimulating hormone (FSH), luteinizing hormone (LH), oestradiol, TSH, ft4, prolactin (PRL), total testosterone, DHEASO4, 17-hydroxyprogesterone (17-OHP), sex-hormone-binding globulin (SHBG), androstendione, 25(OH) vitamin D3 metabolite. The majority of the patients with polycystic ovary syndrome were found to have deficient or suboptimal serum vitamin D levels, and the effects of vitamin D on the SHBG levels and free-androgen indices in these patients was examined. The effects of vitamin D on the incidence of metabolic syndrome and BMI, waist-to-hip ratio, waist circumference, and blood pressure in patients with polycystic ovary syndrome were also found.

## 1. Introduction

Vitamin D, in the form of the active metabolite calcitriol, is a steroidal organic compound whose broad effects on body function are still the subject of numerous clinical studies. Its main function is to regulate calcium-phosphate metabolism and increase the calcium pool through the activation of the RANK/RANKL (receptor activator for nuclear factor κ B/its ligand) pathway in osteoclasts, the stimulation of calcium absorption in the intestine, and its reabsorption in the renal tubules. By binding to the VDR receptor present in immune cells, calcitriol increases the expression of more than 200 genes [1], demonstrating a link between the disturbance of immune homeostasis caused by vitamin D deficiency and the occurrence of autoimmune diseases, such as type I diabetes [2]. Furthermore, a correlation between reduced calcitriol levels and the development of colorectal, breast, and lung cancers has been demonstrated [3]. Moreover, decreased serum vitamin D3 levels are associated with a higher incidence of upper-respiratory-tract infections, chronic obstructive pulmonary disease, and allergic asthma [4]. When used among a population of patients suffering from metabolic syndrome, vitamin D supplementation initiated a marked improvement in tissue insulin sensitivity [5] and also reduced the risk of progression of pre-diabetic conditions, such as abnormal glucose tolerance and abnormal fasting glucose, to type 2 diabetes [6]. It also had an effect on lowering the blood pressure of the aforementioned pool of patients through the inhibition of gene expression for renin [7]. Patients with polycystic ovary syndrome present lower serum vitamin D concentrations compared to the general population. These parameters are even lower in women struggling with obesity [8]—although adipocytes show a high expression of the VDR receptor, fat-soluble vitamin D is retained within adipose tissue, preventing its conversion to a biologically active form [9]. The main natural source of vitamin D is dermal synthesis by sunlight. Exposure of 18% of the body surface without the use of UVA/UVB filters during the hours of maximum sunlight, i.e., between 10 a.m. and 3 p.m., for 15–20 min, should be sufficient for optimal vitamin D synthesis under Polish conditions. However, in our country, vitamin D deficiency occurs at a level of 90% in all age groups, including children [10]. The main purpose behind this study is not only to estimate the serum level of vitamin D 25(OH) in patients with PCOS but also to evaluate its influence on the occurrence of the metabolic syndrome and its components.

## 2. Materials and Methods

### 2.1. Participation in the Study

This study included 120 women aged 18 to 42 years residing in the West Pomeranian Voivodeship who were hospitalized in the Department of Gynaecology, Endocrinology and Gynaecology Oncology, Pomeranian Medical University, in Szczecin, between 2018 and 2021. In our study, patients were tested throughout the year. Due to limited sunny days in Poland, even during summer season, we considered examined women with pcos as having low sun exposure. All women were in the early folliculotropic phase during the study, between days 2 and 5 of their cycle. Patients were divided into two groups:

1. women diagnosed with polycystic ovary syndrome (80 patients)—PCOS group.
2. women menstruating regularly, without features of androgenization, in whom polycystic ovary syndrome was excluded (40 patients).

Exclusion criteria were pregnancy, breastfeeding, thyroid disease, previously diagnosed diabetes, haematological diseases, Cushing’s syndrome, congenital adrenal hyperplasia, fever, and viral and bacterial diseases. The patients were not taking hormonal contraception or hypoglycaemic drugs, nor were they supplementing with vitamin D3.

The Rotterdam ESHRE/ASRM criteria were used to make the diagnosis of polycystic ovary syndrome. Metabolic syndrome was diagnosed using the JIS criteria, the modified IDF 2009 criteria. The study was approved by the Bioethics Committee of the Pomeranian Medical University, number KB-0012/01/18.

### 2.2. Research Methodology

#### 2.2.1. Anthropometric Measurements and Gynaecological Examination

The examinations were performed during hospitalisation in the Department of Gynaecology, Endocrinology and Gynaecological Oncology, Pomeranian Medical University, in Szczecin.

For each patient, the following were performed: history and physical examination, including gynaecological examination—two-handed and in speculum—and ultrasound examination with a vaginal probe to assess the reproductive organs. In the ultrasound examination, ovarian morphology was assessed, measured in three planes, and ovarian volume was calculated by the Voluson P6 software. The severity of hirsutism on the Ferriman–Gallwey scale and the presence of acne and androgenetic alopecia were assessed. In addition, anthropometric measurements were taken—height, weight, waist, and hip circumference were assessed, and blood pressure was measured using the Korotkow method. Patients had their BMI (kg/m^2^) calculated using the following criteria: underweight <18.5, optimal body weight 18.5–24.99, overweight 25.0–29.99, obesity of the first degree 30.0–34.99, obesity of the second degree 35.0–39.99, obesity of the third degree ≥40. Hirsutism was diagnosed when >7 points were obtained on the modified Ferriman–Gallwey scale.

Blood pressure was measured using a mercury-pressure gauge in the sitting position, wearing a cuff sized to the width of the arm 2–3 cm above the elbow flexion. Waist circumference was measured at the end of exhalation at the narrowest point between the upper edge of the iliac crest and the lower edge of the ribcage. Hip circumference was measured at the level of the largest buttock protrusion.

#### 2.2.2. Laboratory Tests

From fasting patients on days 2–5 of their cycle, 5 mL of blood was drawn from the ulnar vein in the morning. In the blood sample obtained, the following parameters were determined: FSH, LH, oestradiol, TSH, ft4, PRL, total testosterone, DHEASO4, 17-OHP, SHBG, androstendione, vitamin D3 metabolite 25(OH). 

The laboratory where the determination of the above parameters was performed adopted the following norms for phase I of the cycle: FSH—3.5–12.5 mlU/mL, LH—2.4–12.6 mlU/mL, oestradiol—12.5–166 pg/mL, TSH—0.51–4.3 ulU/mL, ft4—0.9317 ng/dL, PRL—4, 79–23.3 ng/mL, testosterone—0.06–0.82 ng/mL, DHEASO4—15–19 years—65.1–368 μg/dL, 20–24 years—148–407 μg/dL, 25–34 years—98, 8–340 μg/dL, 35–44 years—80.93370 μg/dL, 17-OHP—0.1–0.8 ng/mL, SHBG—32.4–128 nmol/L, androstendione—0.4–3.4 ng/mL, vitamin D metabolite 25(OH)—<20 ng/mL—deficiency, 20–30 ng/mL—suboptimal concentration, 30–50 ng/mL—optimal concentration, >100 ng/mL—toxic concentration. The FAI was calculated based on the following formula: FAI = total testosterone concentration (nmol/L)/SHBG (nmol/L) × 100%. Values < 5 were considered normal. Determination of vitamin D (25(OH) metabolite) and androstendione was performed by chemiluminescence assay (CLIA) on a LIAISON XL instrument from DiaSorin. Determination of 17-OHP was performed by immunoenzymatic method, ELISA on a Biotek ELX 800 reader. The FSH, LH, oestradiol, prolactin, testosterone, SHBG, DHEASO4, TSH, and ft4 were determined by electrochemiluminescence (ECLIA) on a Cobas PRO module e801 instrument.

### 2.3. Statistical Analysis

The collected data were characterised using descriptive statistics (mean, median, standard deviation, minimum, and maximum values). The assumption of normality of the distribution was checked using the Shapiro–Wilk test. Intergroup comparisons of the collected data were performed using Mann–Whitney U. Correlation analysis was performed using Spearman’s R correlation coefficient. Logistic regression and ROC-curve parameters were calculated to estimate the predictive values of the collected variables on the onset of metabolic syndrome.

## 3. Results

### 3.1. Characteristics of the Group

The study included 80 women with PCOS aged between 18 and 42 years (mean age 26.3 ± 5.1 years) and 40 control women aged between 18 and 46 years (mean age 27.1 ± 6.3 years). There were no significant age differences between the groups (*p* = 0.819).

In the group of women diagnosed with polycystic ovary syndrome, metabolic syndrome was found in 21 patients, representing 26.3% of the group. In the control group, metabolic syndrome was diagnosed in three women, representing 7.5% of the group. The differences between the groups were significant (*p* = 0.015) (Table 1).

### 3.2. Comparison of Anthropometric Results in the Female Subjects

The mean BMI among the women in the PCOS group was 27.26 kg/m^2^, SD 5.46. In the control group, the mean value was 23.58 kg/m^2^, SD 4.41. The BMI values were found to be significantly higher in the PCOS group compared with the control group *p* = 0.000. In the PCOS group, 26.3% (*n* = 21) of the women were overweight, 18.8% (*n* = 15) were grade I obese, 8.8% were grade II obese, and 2.5% (*n* = 2) of the women were grade III obese. In the control group, 77.5% (*n* = 31) of the women studied were of normal weight, 15% (*n* = 6) were overweight, and 2.5% (*n* = 1) each had grade I, II, and III obesity, respectively. The mean waist circumference in the group of women with PCOS was 87.08 cm, SD 17.1, while in the control group, it was 76.28 cm, SD 12.84. The waist-circumference values were found to be significantly higher in the patients with PCOS compared with those in the control group *p* = 0.001. Abdominal obesity was found in 62.5% (*n* = 50) of the women with PCOS, while in the control group, a waist circumference >80 cm was found in 27.5% (*n* = 11).

The mean waist-to-hip ratio (WHR) was 0.83, SD 0.12 in the group of women with PCOS, while in the control group, it was 0.76, SD 0.08. The differences between the groups were significant, with *p* = 0.001 (Table 2).

The mean value of diastolic blood pressure in the women with PCOS was 79 mmHg, SD 11.64. In the control group, the mean value was 77 mmHg, SD 8.26. The differences between the groups were significant, with *p* = 0.044. The mean systolic blood pressure in the women with PCOS was 125 mmHg, SD 11.87. In the control group, the mean value was 117 mmHg, SD 12.9. Significantly higher systolic-blood-pressure values were found in the group of patients with PCOS compared to the control group *p* = 0.001. Blood pressure >130/85 mmHg was diagnosed in 48.8% (*n* = 39) of the patients with PCOS and 20% (*n* = 8) of the patients in the control group (Table 3).

### 3.3. Comparison of Concentrations of Selected Hormones in the Women Studied

The mean LH concentration in the group of women with PCOS was 9.68 nU/mL, SD 5.08, while in the control group, the mean value was 6.03 nU/mL, SD 3.28. The differences between the groups were significant, with *p* = 0.000.

The mean testosterone concentration in the group of women with PCOS was 0.5 ng/mL, SD 0.22, while in the control group, the mean value was 0.35 ng/mL, SD 0.15. The differences between the groups were significant, with *p* = 0.000. The mean value of the 17(OH)P concentration in the group of women with PCOS was 1.22 ng/mL, SD 0.42, while in the control group, the mean value was 0.96 ng/mL, SD 0.37. The differences between the groups were significant *p* = 0.001 (Table 4).

The mean SHBG-concentration value in the group of women with PCOS was 51.6 nmol/L, SD 34.35, while in the control group, the mean value was 87.6 nmol/L, SD 54.25. The differences between the groups were significant *p* = 0.000.

The mean androstendione value in the group of women with PCOS was 3.31 ng/mL, SD 1.48, while in the control group, the mean value was 2.1 ng/mL, SD 0.54. The differences between the groups were significant, with *p* = 0.000. The mean FAI value in the group of women with PCOS was 4.97, SD 3.79, while in the control group, the mean value was 1.74, SD 0.98. The differences between the groups were significant *p* = 0.000.

There were no statistically significant differences in the concentrations of oestradiol, FSH, prolactin, DHEASO4, TSH, and ft4 between the compared groups (Table 5).

### 3.4. Vitamin D 25(OH)

The mean vitamin D concentration (25(OH-metabolite) in the group of women with PCOS was 22.44 ng/mL, SD 11.84. In the control group, the mean value was 24.62 ng/mL, SD 10.38. The higher mean vitamin D concentration in the group of patients with PCOS was not statistically significant *p* = 0.238 (Table 6).

In the PCOS group, 42.5% (*n* = 34) of the women had vitamin D deficiency, 35% (*n* = 28) had suboptimal levels, and 22.5% (*n* = 18) had optimal levels. In the control group, vitamin D deficiency was found in 35% (*n* = 14), suboptimal levels were found in 45% (*n* = 18), and optimal levels were found in 20% (*n* = 8) of the women (Table 7).

In the PCOS group, a statistically significant negative correlation was observed between the serum vitamin D 25(OH) levels and the occurrence of the metabolic syndrome (R = −0.30; *p* = 0.008), BMI (R = −0.27; *p* = 0.014), waist circumference (R = −0.27; *p* = 0.014), WHR (R = −0.23; *p* = 0.042), FAI coefficient (R = −0.29; *p* = 0.008), systolic blood pressure (R = −0.30; *p* = 0.006), and diastolic blood pressure (R = −0.27; *p* = 0.015). A statistically significant positive correlation was observed between the serum vitamin D 25(OH) levels and the SHBG (R = 0.36; *p* = 0.001) levels (Table 8).

In the control group, there were no statistically significant correlations between the vitamin D 25(OH) concentrations and the parameters shown in the table.

A univariate logistic regression analysis was performed for vitamin D, where the dependent variable was the metabolic syndrome. The odds ratio for vitamin D was 0.93, meaning that a unit increase in vitamin D reduces the odds of the metabolic syndrome by 7% (Table 9).

**Figure 1:**
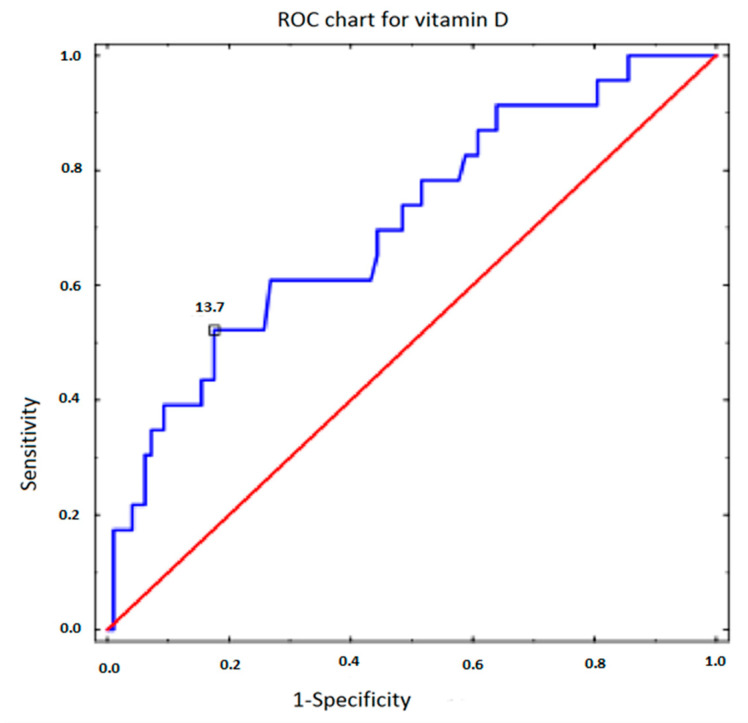
The ROC curve for vitamin D.

A receiver operating Characteristic (ROC) analysis of vitamin D was performed, in which the dependent variable was metabolic syndrome (Figure 1). The cut-off points were determined for the parameters in which the sensitivity and specificity were most optimal. The sensitivity represented the percentage of patients with metabolic syndrome who were correctly diagnosed based on the level of vitamin D concentration. The specificity represented the percentage of people correctly classified as healthy who did not have the metabolic syndrome. The classification quality of the parameters was determined by the AUC. The closer the AUC value was to unity, the better the predictive power of the variable under study. For vitamin D, the cut-off point was 13.7 (ng/mL). The sensitivity for the cut-off point was 0.522 and the specificity was 0.825. The area under the curve (AUC) was 0.706 (*p* = 0.001) (Table 10).

## 4. Discussion

The main objective of this study was to assess the serum level of vitamin D 25(OH) in patients with PCOS and to evaluate its impact on the occurrence of the metabolic syndrome and its components. The definition of the metabolic syndrome encompasses the presence of obesity and two of the three following criteria: hypertension, impaired glucose metabolism, and elevated non-HDL-cholesterol levels (atherogenic dyslipidaemia). The association of vitamin D deficiency with the development of the metabolic syndrome, hypertension, insulin resistance, and obesity, disease entities that very often coexist with polycystic ovary syndrome, has been demonstrated [11,12].

In our study, there were no significant differences between the serum vitamin D concentrations in the patients with polycystic ovary syndrome and those in the control group (*p* = 0.238). Although no significant differences were found between the groups, it should be noted that vitamin D deficiency was found in as many as 42.5% of the women in the PCOS group, while suboptimal concentrations were found in 35%, and normal concentrations were found in only 22.5% of the subjects. However, many authors have obtained results that contradict those of our study, showing that patients with polycystic ovary syndrome have lower vitamin D concentrations than those in the general population [13,14].

In conclusion, the differences between the findings of many authors on vitamin D levels in patients with PCOS are inconclusive for a number of reasons. First of all, the studies adopted different diagnostic criteria for polycystic ovary syndrome—not all the studies used the Rotterdam criteria, which may have affected the final results. Furthermore, studies were conducted in different geographical regions. Although vitamin D deficiency is a global problem, significant differences in mean 25(OH)D concentrations between populations can be observed. In addition, a higher proportion of the population in developed countries orally supplement vitamin D than in developing countries. The vitamin D concentrations in the study groups were also influenced by the time of year of determination. The vast majority of previous studies did not take this factor into account. In our study, the patients were tested throughout the year.

A significant relationship between vitamin D and body weight has been demonstrated, with values significantly lower in obese individuals. In most of the studies available in the literature, but also in our own study, the groups of patients with PCOS and the control groups were not similar in terms of BMI, which may have affected the results obtained.

In our study on patients with PCOS, vitamin D 25(OH) showed a significant negative correlation with the metabolic syndrome, BMI, WHR, waist circumference, and FAI index. Vitamin D showed a positive correlation with SHBG. These results are consistent with the data reported in the literature.

According to our research, more than half of the patients with PCOS had a BMI > 25 kg/m^2^. Most authors agree on the association of vitamin D deficiency with excess body weight. Muscogiuri et al. [15] determined body-fat content in PCOS patients through X-ray absorptiometry and demonstrated that serum 25(OH)D levels are closely related to body-fat percentage. The main hypothesis explaining these reports is that vitamin D, as a fat-soluble substance, is retained in adipose tissue, preventing its conversion to active metabolites [16]. In addition, obese people are thought to have less exposure to sunlight compared to physically active people of normal weight. The correlation of vitamin D with BMI and a higher percentage of 25(OH)D deficiency among obese PCOS patients was demonstrated by Panidis et al. [17], Savastano et al. [18], Hahn et al. [19], and Tsakova et al. [20].

Our own study showed a negative correlation between 25(OH)D and blood pressure. Furthermore, Lind et al. [21] showed a reduction in mean blood-pressure concentrations in patients after 18 months of vitamin D supplementation.

Hyperandrogenaemia is one of the diagnostic criteria for polycystic ovary syndrome and affects 60–80% of patients. There are conflicting reports on the effects of vitamin D on androgen levels in patients with PCOS. Our study showed a correlation of 25(OH)D with FAI and SHBG. Muscogiuri et al. [11] made similar observations in their study on a group of women with PCOS in the Italian population. Hahn et al. [15] demonstrated an association of serum vitamin D levels with the severity of hirsutism in patients with PCOS. He et al. [8], in a large meta-analysis, also showed a correlation between vitamin D, FAI, and SHBG, which is consistent with the results obtained in our study. However, Zhao et al. [22] did not prove an association of vitamin D supplementation with improvements in the hormonal profiles of PCOS patients.

Our study on patients with polycystic ovary syndrome showed an association of vitamin D with two of the five diagnostic criteria for the metabolic syndrome—waist circumference and blood pressure. Furthermore, a negative correlation with the presence of the metabolic syndrome in these patients was shown (r = −0.30; *p* = 0.008). In the control group, vitamin D showed no correlations with any of the parameters determined.

In 2005, Ford et al. [23], published a paper linking vitamin D deficiency to the incidence of the metabolic syndrome in the general population. Wehr et al. [24], studying more than 200 patients diagnosed with PCOS based on the Rotterdam criteria, obtained results consistent with those of Ford et al.’s study, highlighting the impact of vitamin D deficiency on the development of the metabolic syndrome and its components.

In a univariate logistic regression analysis of vitamin D, it was shown that a unit increase in vitamin D reduces the odds of the metabolic syndrome in patients with PCOS by 7%. Furthermore, Akbari et al. [25] and Mansournia et al. [26], in their meta-analyses, pointed to the beneficial effect of vitamin D supplementation on the reduction in inflammation parameters. Therefore, it may seem reasonable to supplement these patients with vitamin D to reduce their risk of developing the metabolic syndrome and cardiovascular complications.

Unfortunately, the crucial role of meaningful supplementation tends to be forgotten—the main idea behind our study is to consider it as a therapeutic approach rather than a form of daily self-care and engage patients to engage in the treatment process by offering them a form of medicine that is easy for them to access. The positive effects listed above are clear.

## 5. Conclusions

There were no significant differences in serum vitamin D levels between the patients with polycystic ovary syndrome and the controls; most of the patients with polycystic ovary syndrome showed deficient or suboptimal serum vitamin D levels.

Although our study did not find any significant differences in vitamin D levels between women with and without PCOS, it established that patients with polycystic ovary syndrome have a significantly higher incidence of the metabolic syndrome and MI, as well as a higher waist-to-hip ratio, waist circumference, and blood pressure. Furthermore, PCOS is associated with lower levels of SHBG and higher levels on the free-androgen index.

No correlations were found between vitamin D levels and FSH, LH, PRL, oestradiol, testosterone, 17(OH)P, DHEASO4, androstendione, tsh, or ft4 in patients with polycystic ovary syndrome. 

## Figures and Tables

**Table 1:** Prevalence of metabolic syndrome in the study groups of women. Comparison *p*-values between the PCOS group (*n* = 80) and the control group (*n* = 40).

	*n*	%	*p*
PCOS	21	26.3	0.015
Control	3	7.5	

**Table 2:** Characteristics of anthropometric parameters. Comparison of *p*-values between the PCOS group (*n* = 80) and the control group (*n* = 40).

Group	Median	Q1	Q3	*p*
Waist circumference (cm)				
PCOS	85.50	71.50	100.50	0.001
Control	72.00	68.00	81.50	
WHR				
PCOS	0.82	0.73	0.89	0.001
Control	0.74	0.70	0.81	
BMI (kg/m^2^)				
PCOS	26.05	17.70	42.00	0.000
Control	22.65	18.80	41.20	
Systolic pressure (mmHg)				
PCOS	125.50	119.00	133.50	0.001
Control	119.00	110.00	125.50	
Diastolic pressure (mmHg)				
PCOS	81.00	72.00	86.00	0.044
Control	76.00	72.00	81.50	

**Table 3:** Prevalence of deviations in anthropometric parameters. The *p*-values of comparisons between the PCOS group (*n* = 80) and the control group (*n* = 40) are shown.

	PCOS		Control		
Waist circumference	*n*	%	*n*	%	*p*
<80 cm	30	37.5	29	72.5	0.000
>80 cm	50	62.5	11	27.5	
BMI					
<18.5 kg/m^2^	1	1.3	0	0	0.011
18.5–24.99 kg/m^2^	34	42.5	31	77.5	
25.0–29.99 kg/m^2^	21	26.3	6	15.0	
30.0–34.99 kg/m2	15	18.8	1	2.5	
35.0–39.99 kg/m^2^	7	8.8	1	2.5	
>40.0 kg/m^2^	2	2.5	1	2.5	
Blood pressure					
<130/85 mmHg	41	51.3	32	80.0	0.002
≥130/85 mmHg	39	48.8	8	20.0	

**Table 4:** Characterisation of concentrations of selected hormones. The *p*-values of comparisons between the PCOS group (*n* = 80) and the control group (*n* = 40) are shown.

	Group	Median	Q1	Q3	*p*
TSH (mU/mL)	PCOS	1.69	1.18	2.28	0.413
	Control	1.59	1.17	2.05	
FT4 (ng/dL)	PCOS	1.19	1.02	1.31	0.228
	Control	1.22	1.03	1.34	
PRL (ng/mL)	PCOS	10.60	7.66	16.61	0.978
	Control	10.85	8.21	16.55	
FSH (mU/mL)	PCOS	5.96	4.58	6.80	0.399
	Control	5.49	4.48	6.54	
LH (mU/mL)	PCOS	8.56	6.07	13.01	0.000
	Control	6.09	4.40	8.22	
E2 (ng/mL)	PCOS	40.95	30.33	51.90	0.709
	Control	41.55	27.75	65.37	
T (ng/mL)	PCOS	0.43	0.32	0.66	0.000
	Control	0.34	0.26	0.43	
DHEAS04 (μg/dL)	PCOS	253.00	199.50	358.00	0.184
	Control	244.50	185.00	298.40	
17(OH)P (ng/mL)	PCOS	1.10	0.91	1.51	0.001
	Control	0.91	0.73	1.09	
SHBG (nmol/L)	PCOS	38.56	28.20	68.90	0.000
	Control	70.95	52.70	107.60	
Androstendione (ng/mL)	PCOS	2.99	2.21	3.95	0.000
	Control	2.19	1.96	2.39	
FAI	PCOS	4.34	2.03	6.57	0.000
	Control	1.85	0.75	2.32	

**Table 5:** Prevalence of deviations in concentrations of selected hormones. The *p*-values of comparisons between the PCOS group (*n* = 80) and the control group (*n* = 40) are shown.

	PCOS		Control		
TSH	*n*	%	*n*	%	*p*
<0.51 mU/mL	1	1.3	2	5.0	0.285
0.51–4.3 mU/mL	77	96.3	38	95.0	
>4.3 mU/mL	2	2.5	0	0	
FT4	n	%	n	%	
<0.93 ng/dL	12	15.0	3	7.5	0.411
0.93–1.7 ng/dL	66	82.5	35	87.5	
>1.7 ng/dL	2	2.5	2	5.0	
PRL	n	%	n	%	
<4.79 ng/mL	3	3.8	3	7.5	0.247
4.79–23.3 ng/mL	69	86.3	36	90.0	
>23.3 ng/mL	8	10.0	1	2.5	
FSH	n	%	n	%	
<3.5 mU/mL	6	7.5	5	12.5	0.371
3.5–12.5 mU/mL	74	92.5	35	87.5	
LH	n	%	n	%	
<2.4 mU/mL	4	5.0	6	15.0	0.002
2.4–12.6 mU/mL	55	68.8	33	82.5	
>12.6 mU/mL	21	26.3	1	2.5	
E2	n	%	n	%	
<12.5 ng/mL	2	2.5	3	7.5	0.375
12.5–166 ng/mL	77	96.3	36	90.0	
>166 ng/mL	1	1.3	1	2.5	
T	n	%	n	%	
<0.06 ng/dL	0	0	1	2.5	0.034
0.06–0.82 ng/dL	71	88.8	39	97.5	
>0.82 ng/dL	9	11.3	0	0	
DHEA SO4	n	%	n	%	
Below normal (for age)	0	0	1	2.5	0.011
Standard (for age)	67	83.8	39	97.5	
Above standard (for age)	13	16.3	0	0	
17(OH)P	n	%	n	%	
0.1–1.4 ng/mL	56	70.0	36	90.0	0.014
>1.4 ng/mL	24	30.0	4	10.0	
SHGB	n	%	n	%	
<32.4 nmol/mL	30	37.5	0	0	0.000
32.4–128 nmol/mL	46	57.5	34	85.0	
>128 nmol/mL	4	5.0	6	15.0	
Androstendione	n	%	n	%	
0.4–3.4 ng/mL	54	67.5	40	100	0.000
>3.4 ng/mL	26	32.5	0	0	

**Table 6:** Characterisation of vitamin D 25(OH) concentrations. The *p*-values of the comparisons between the PCOS group (*n* = 80) and the control group (*n* = 40) are shown.

Vitamin D 25(OH) (ng/mL)	Group	Median	Q1	Q3	*p*
	PCOS	22.50	11.95	28.95	0.238
	Control	23.45	16.30	29.15	

**Table 7:** Prevalence of deviations in vitamin D 25(OH) concentration. The *p*-values of comparisons between the PCOS group (*n* = 80) and the control group (*n* = 40) are shown.

	PCOS		Control		*p*
Vitamin D 25(OH)	%	*n*	%	*n*	0.563
<20 ng/mL	42.5	34	35	14	
20–30 ng/mL	35	28	45	18	
30–50 ng/mL	22.5	18	20	8	

**Table 8:** Correlation analysis of vitamin D levels with metabolic-syndrome prevalence, hormones, and anthropometric parameters in the PCOS group.

	PCOS		Control	
Parameter	R Spearmana	*p*	R Spearmana	*p*
Metabolic syndrome	−0.30	0.008	−0.24	0.138
TSH (mU/mL)	−0.02	0.871	0.00	0.992
FT4 [(ng/dL)	0.02	0.876	0.04	0.806
PRL (ng/mL)	−0.21	0.058	−0.04	0.815
FSH (mU/mL)	0.03	0.761	−0.02	0.913
LH (mU/mL)	0.03	0.780	−0.07	0.686
E2 (ng/mL)	−0.05	0.688	−0.15	0.350
T (ng/mL)	−0.08	0.479	−0.03	0.849
DHEAS04	0.01	0.915	0.08	0.602
17(OH)P (ng/mL)	−0.04	0.706	0.07	0.657
SHBG (nmol/L)	0.36	0.001	−0.25	0.115
Androstendione (ng/mL)	−0.11	0.332	0.04	0.826
FAI	−0.29	0.008	0.05	0.740
Waist circumference	−0.27	0.014	−0.26	0.108
WHR	−0.23	0.042	−0.21	0.198
BMI	−0.27	0.014	−0.17	0.291
Systolic pressure (mmHg)	−0.30	0.006	−0.09	0.592
Diastolic pressure (mmHg)	−0.27	0.015	−0.28	0.079

**Table 9:** Evaluation of vitamin D in univariate logistic regression in relation to metabolic-syndrome in patients with PCOS.

Parameter	Evaluation	OR	−95% CI	95% CI	*p*
Vitamin D	−0.078	0.93	0.88	0.98	0.005

**Table 10:** ROC-curve parameters (sensitivity, specificity, area under the curve (AUC)) for vitamin D in relation to the metabolic syndrome in patients with PCOS.

Parameter	Sensitivity	Specificity	AUC	*p*
Vitamin D	0.522	0.825	0.706	0.001

## Data Availability

Data will be available upon prior request from the first author.
